# Therapeutic Efficacy and Promise of Human Umbilical Cord Mesenchymal Stem Cell-Derived Extracellular Vesicles in Aging and Age-Related Disorders

**DOI:** 10.3390/ijms26010225

**Published:** 2024-12-30

**Authors:** Anyuan Zhang, Qiubai Li, Zhichao Chen

**Affiliations:** 1 Department of Hematology, Union Hospital, Tongji Medical College, Huazhong University of Science and Technology, Wuhan 430022, China; anyuanzhangcm@163.com; 2 Department of Rheumatology and Immunology, Union Hospital, Tongji Medical College, Huazhong University of Science and Technology, Wuhan 430022, China

**Keywords:** extracellular vesicles, umbilical cord, mesenchymal stem cells, anti-aging, age-related disorders

## Abstract

The global issue of aging populations has become increasingly prominent, thus the research and development for anti-aging therapies to assure longevity as well as to ameliorate age-related complications is put high on the agenda. The young humoral milieu has been substantiated to impart youthful characteristics to aged cells or organs. Extracellular vesicles (EVs) are a heterogeneous group of cell-derived membrane-limited structures that serve as couriers of proteins and genetic material to regulate intercellular communication. Of note, EVs appeared to be an indispensable component of young blood in prolonging lifespans, and circulating EVs have been indicated to mediate the beneficial effect of a young milieu on aging. Human umbilical cord mesenchymal stem cell-derived EVs (HUCMSC-EVs), isolated from the youngest adult stem cell source, are speculated to reproduce the function of circulating EVs in young blood and partially revitalize numerous organs in old animals. Robust evidence has suggested HUCMSC-EVs as muti-target therapeutic agents in combating aging and alleviating age-related degenerative disorders. Here, we provide a comprehensive overview of the anti-aging effects of HUCMSC-EVs in brain, heart, vasculature, kidney, muscle, bone, and other organs. Furthermore, we critically discuss the current investigation on engineering strategies of HUCMSC-EVs, intending to unveil their full potential in the field of anti-aging research.

## 1. Introduction

The aging process manifests itself as a complex network of highly interconnected biological cascades leading to progressive degeneration of organs throughout the body. Aging is the primer risk factor for cardiovascular disease, diabetes mellitus, neurodegenerative diseases, and bone and joint disorders [1], and the resulting structural and functional deterioration of tissues and organs constitutes a public hazard in the elderly population. Researchers have long attempted to halt or potentially reverse aging in a bid to reduce clinical burden and extend healthspans [2]. There are several well-established hallmarks of aging that can be generally categorized into three distinct levels based on their origins and mechanisms, including genomic instability and telomere attrition at the molecular level, autophagy dysregulation, mitochondrial dysfunction and cellular senescence at the cellular level, and stem cell exhaustion and chronic inflammation at a systemic level [3]. These hallmarks are interconnected rather than independent, involving intricate molecular, cellular, and systemic interactions that collectively drive the aging process. They also offer valuable insights for the development of therapeutic interventions aimed to decelerate, halt, or reverse aging. Currently, strategies of rejuvenation based on young humoral milieu such as heterochronic parabiosis, neutral blood exchange, young bone marrow transplantation, and young cerebrospinal fluid infusion have been shown to be effective in conferring more youthful characteristics to senescent organs, and delaying the aging process to achieve revitalization [4]. Indeed, numerous studies have shown that exposure to a young systemic environment can reverse cellular senescence and boost longevity in aged mice [5,6,7].

Extracellular vesicles (EVs) are membranous nanostructures secreted by all eukaryotic cells that are widely distributed in biological fluids, with a high degree of structural and functional heterogeneity, and are involved in a wide range of physiological and pathological processes [8,9]. According to Minimal Information for Studies of Extracellular Vesicles (MISEV2023), EVs separated with methods such as filtration or differential ultracentrifugation are recommended to be categorized into large EVs (diameter > 200 nm, including apoptotic vesicles) and small EVs (diameter < 200 nm) based on their diameter size [10]. The specialized term, apoptotic vesicles, refers to a distinctive population of EVs that are generated during cell apoptosis [10,11]. EVs encompass a variety of bioactive molecules, including proteins, lipids, and nucleic acids, and are recognized as a new axis for intercellular communication [12]. The bioinformation harbored in EVs determines the unique functionality and performance of these nanovesicles, reflecting the function and state of producing cells and holding promise to convert recipient cells into their parental cells [13]. Hence, EVs are considered to be of great value for the development of biomarkers and therapeutic drugs for the diagnosis and treatment of diseases [14,15]. More importantly, the dual roles these nanovesicles played in the aging process are now widely recognized [16]. In brief, senescent cells transfer cellular senescence signals by releasing large amounts of pro-inflammatory cytokines and EVs, termed as senescence-associated secretory phenotype (SASP), which are secreted in a paracrine fashion to deliver pro-aging signals leading to the senescence of neighboring normal cells [17]. By contrast, EVs derived from young humoral milieu or isolated from young cells, mainly stem cells, are more than likely to exert the specific rejuvenating effects and delay the aging process, thus showing great potential to mitigate age-related degeneration [18].

Recent research on combating aging by heterochronic parabiosis and young plasma infusion has attributed the rejuvenating effect to the circulating EVs carrying and transferring abundant youthful signals, which are internalized into senescent cells and thus ameliorate the aging-associated phenotypes [7]. The emergence of young blood circulating EVs as rejuvenating effect mediators has been highlighted [19]. Mesenchymal stem cell (MSC) therapy has been the leading framework in regenerative medicine and EVs derived from MSCs have been positioned as potential alternatives with remarkable therapeutic benefits. Statistically, MSCs are the most frequently documented sources in current EV-based clinical studies [20]. In particular, bone marrow, adipose tissue, and umbilical cord are the most commonly utilized sources of MSCs [20]. Notably, the isolation of MSCs from bone marrow and adipose tissue entails a series of complicated tissue collection procedures, which are invasive and painful for patients and pose a risk of infection [21]. As a contrast, the collection of human umbilical cord mesenchymal stem cells (HUCMSCs) is a non-invasive procedure, as umbilical cords are typically regarded as waste products and are usually discarded after birth, thereby circumventing the ethical concerns associated with the utilization of embryonic stem cells and other adult stem cells [21,22]. HUCMSCs, as the youngest primitive adult stem cell, have not been affected by any acquired disorders and have the fastest population doubling time, and are consequently identified as promising seeding cells and an optimal cell source for stem cell-based and EV-based anti-aging therapies [23,24]. As a corollary, HUCMSC-derived EVs (HUCMSC-EVs), which share similar functions to HUCMSCs and exhibit superior immunomodulatory effects compared to EVs from alternative sources [25], have been extensively investigated in the anti-aging field, and are highly likely to serve as a competent alternative to young blood circulating EVs in the heterochronic parabiosis model. In this review, we summarize the research progress of HUCMSC-EVs in the anti-aging domain, delineate their unique rejuvenating effects in age-related degeneration and diseases in brain, heart, vasculature, kidney, bone, muscle, and other organs, and discuss the striking properties and underlying mechanisms of their anti-aging effect. We also outline the current efforts to engineer HUCMSC-EVs to unlock their full therapeutic potential as next-generation cell-free anti-aging biotherapeutics.

## 2. The Expanding Universe of HUCMSC-EVs: An Overview

EVs from the plasma of young mice have been shown to counteract senescence at the molecular, cellular, and physiological levels [26,27,28,29]. Young blood-derived EVs appear to be a beneficial component that may mediate the aging process through non-cell-autonomous mechanisms and explain the rejuvenating effect of young circulation [7]. Direct isolation of circulating EVs from the blood of young individuals faces challenges such as lack of standard acquisition and extraction protocols, strong heterogeneity, inconsistent identification criteria, and inevitable ethical issues [30]. Consequently, it is necessary and imperative to capitalize on EVs derived from young cells that can be expanded in vitro on a large scale to reproduce the anti-aging effect and further explore the molecular targets.

Umbilical cord is the optimal cell source on account of its available and noninvasive collection, and the MSCs from this source are more capable and prolific [31]. HUCMSCs have superior multipotentiality and protective effects and simultaneously avoid ethical dilemmas compared with other kinds of stem cells [32,33]. The therapeutic value, current challenges, and future directions for the clinical application of HUCMSCs have been fully evaluated [32,34]. Of note, there is an inextricable relationship between the rejuvenating effects of HUCMSCs and the secretion of active compounds that may be packaged in EVs [35,36], thus supporting rigorous investigation on HUCMSC-EVs as a theoretical alternative to young blood circulating EVs. Currently, the predominant technique for isolating HUCMSC-EVs remains ultracentrifugation of the supernatant obtained from in vitro cultured HUCMSCs [37]. Due to the absence of definitive markers, the overall characterization of HUCMSC-EVs in most studies generally depends on identifying canonical exosomal surface markers such as CD63, CD9, CD81, and TSG101 through Western Blot, along with the detected multipotent differentiation potential of their parental cells [38]. Additionally, the size distribution and morphological features are usually analyzed using imaging techniques like transmission electron microscopy (TEM) and scanning electron microscopy (SEM), as well as biophysical methods including nanoparticle tracking analysis (NTA) and flow cytometry, which provide comprehensive profiling of single HUCMSC-EV [10].

HUCMSC-EVs embody both the immunomodulatory and tissue-repairing capacity possessed by HUCMSCs and inherent signaling carrier properties of EVs, thereby demonstrating extraordinary regulatory properties in inflammation, apoptosis, autophagy, and oxidative stress by virtue of bioactive cargos. Meanwhile, HUCMSC-EVs have numerous advantages including lower immunogenicity, higher biological stability, better bioavailability, easier access, and freedom from tumorigenic potential and ethical issues over their parental cells [39]. Therefore, HUCMSC-EVs are described as a viable cell-free therapy in managing various pathologies including inflammatory diseases [40,41,42], allergic diseases [43], obstetric diseases [44], autoimmune diseases [45,46], and tumors [47,48]. More importantly, mounting evidence has showcased the salient geroprotective features of HUCMSC-EVs in age-related degeneration and diseases encompassing Alzheimer’s diseases, Parkinson’s diseases, myocardial infarction, atherosclerosis, chronic kidney disease, sarcopenia, osteoporosis, osteoarthritis, and diabetes (detailed below). In general, HUCMSC-EVs not only inhibit the loss of functional parenchymal cells, but also promote the proliferation and migration of interstitial cells to maintain the structural and functional integrity of aged or injured tissues. Mechanistically, HUCMSC-EVs can deliver bioactive cargos to areas needing regeneration to maintain tissue viability in age-related complications. By virtue of these nanostructures, abundant youthful signals complete the whole journey from HUCMSCs (the youngest adult stem cell type) to senescent recipient cells. Anti-aging proteins, miRNAs, etc., are internalized into senescent cells and act on downstream pathways, targeting hallmarks of aging, such as genomic instability, telomere attrition, mitochondrial dysfunction, and senescence-associated secretory phenotypes [3], thus efficiently improving multiple senescence-associated phenotypes. In summary, HUCMSC-EVs are a group of unique, cargo-bearing and multi-target particles, and unveiling their anti-aging potential is conducive to the proposal and development of new strategies to combat the aging process.

## 3. The Therapeutic Efficacy of HUCMSC-EVs in Counteracting Multi-Organ Aging

**Figure 1:**
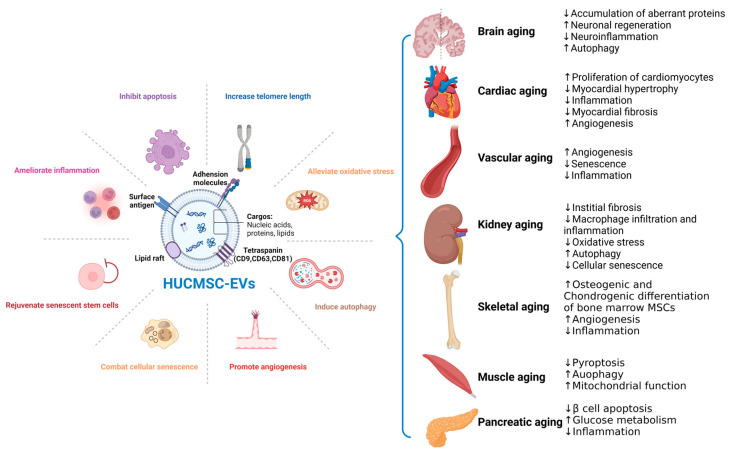
Schematic showing the biological structure and properties of HUCMSC-EVs and their therapeutic effects in aging across multiple organs. HUCMSC-EVs are nanostructures encapsulated by a bilayer lipid membrane and contain an extensive array of bioactive molecules. They engage in various pathophysiological processes and possess multifaceted regenerative properties to mitigate age-related disorders in multiple organs. For each condition, the observed impacts of HUCMSC-EVs on various biological processes are illustrated, with arrows denoting enhancements or reductions in specific processes. Created with BioRender.com (accessed on 24 November 2024).

Similar to circulating EVs from young blood, HUCMSC-EVs have served as a multi-target cell-free biotherapeutics of age-related disorders in preclinical animal models (See Figure 1).

### 3.1. Effect of HUCMSC-EVs on Brain Aging

Brain aging is manifested by alterations in brain morphology, pathological accumulation of aberrant proteins, and maladjusted physiological functions, which dramatically increases the risks of a range of neurodegenerative diseases [49]. A vast body of evidence suggests that the brain is highly susceptible to the rejuvenation of young blood, and various cell types in brain exhibit obvious plasticity after exposure to young circulation [6,50]. Consistently, beneficial effects of HUCMSC-EVs to restore neurological dysfunction by acting on neurons and glial cells were displayed in the context of trauma [51] and age-related neurodegenerative pathologies [52]. Specifically, HUCMSC-EVs can inhibit the apoptosis and promote the regeneration of neurons [53], and simultaneously have extensive cross-talks with glial cells [54], thus attenuating the neuroinflammation especially triggered by the microglial activation. Hereinafter, we put emphasis on the therapeutic potential of HUCMSC-EVs for Alzheimer’s disease and Parkinson’s disease.

Alzheimer’s disease (AD) is the most common neurodegenerative disease characterized by synaptic deficits and neuronal loss due to excessive accumulation of the amyloid-β peptide (Aβ) in brain tissues, which has been considered as the root cause of neuroinflammation [55]. The core pathophysiology lays a foundation for the therapeutic efficacy of HUCMSC-EVs in AD models, illustrated by the restoration of memory and cognitive dysfunction [56,57]. Mechanistically, it was found via bioinformatics that adaptor-related protein complex 2 subunit alpha 1 (AP2A1) and subunit beta 1 (AP2B1) in HUCMSC-EVs played significant roles in modulating the expression of neuronal memory/synaptic plasticity-related genes through the synaptic vesicle cycle signaling pathway [58]. Of note, Neprilysin (NEP) is a main cleavage enzyme of Aβ, and increasing NEP expression in AD animal models has an ameliorative effect [59,60]. NEP-enhanced HUCMSCs had superior neurogenesis and anti-inflammation properties due to increased NEP in the hippocampus by enriched NEP-possessing EVs [61]. Concurrently, HUCMSC-EVs overexpressing miR-211-5p (a potent repressor of NEP expression) inhibitor exhibited greater efficiency in mitigating the injury caused by Aβ_1–40_ treatment [62]. Interestingly, EVs derived from HUCMSCs cultured in the 3D graphene scaffold showed a stark enhancement in reducing the production of Aβ in AD pathological cells and transgenic mice, and restoring the memory and cognitive deficits of AD mice through their altered cargo compositions [63]. In the future, it is of prime interest to discover more effector molecules in HUCMSC-EVs that contribute to clearing Aβ accumulated pathologically.

Age is the most significant risk factor for Parkinson’s disease (PD), which is the second most common progressive neurodegenerative disorder and featured with motor deficits due to the depletion of nigrostriatal dopamine [64]. In recent years, the use of MSCs for the treatment of PD has expanded to supplement and rescue dopaminergic neurons, thus alleviating locomotor deficits [65,66,67]. Similarly, it has been reported that EVs from HUCMSCs mitigated neuronal damage and upregulated the level of dopamine for PD treatment via upregulation of special AT-rich sequence-binding protein-1 (SATB 1) and activation of Wnt/β-catenin pathway to modulate autophagy [68,69]. The glial cell-mediated inflammatory response plays a central role in Parkinson’s disease, and therefore, the capacity of HUCMSC-EVs to relieve neuroinflammation in the treatment of PD models comes to the fore. HUCMSC-EVs administered through intranasal, tail vein, or lateral ventricle injection inhibited microglia and astrocyte activation to relieve neuroinflammation [70,71], which can be explained by the enrichment of miRNAs (miR-7, miR-125-5p, miR-122-5p, miR-126-3p, and miR-199-3p, etc.) and proteins in some signaling pathways related to inflammation or immunity (leukocyte trans-endothelial migration, PI3K/AKT, etc.) [71]. Another innovative study reported that anti-amyloid drugs delivered by nanoliposome in combination with HUCMSC-EVs may help to protect vulnerable neurons by preventing and reversing amyloid formation (as a marker for the progression of PD), aided by the HUCMSC-EVs’ ability to cross the blood–brain barrier and increase the internalization of drugs [72].

### 3.2. Effect of HUCMSC-EVs on Cardiac Aging

Cardiac aging is evident by diminished regenerative capability which subsequently increases susceptibility to heart failure induced by ischemic injury. Given the limited self-renewal rate of cardiomyocytes in the adult heart, when myocardial apoptosis induced by injuries occurs during aging, the surviving cardiomyocytes develop compensatory hypertrophy, with excessive proliferation of fibroblasts and inflammatory cells to maintain structural integrity [49]. The aged heart exhibits accumulated byproducts of aerobic metabolism and lipids, with concomitant cardiac hypertrophy and diffuse fibrosis, therefore leading to cardiac remodeling and dysfunction [73]. There is an urgent need to search for effective rejuvenating agents to counteract cardiac aging.

Repeated administration of EVs secreted by young cardiosphere-derived cells in aged rodents triggered stark functional enhancement, especially in the aged heart, with concordant structural changes and robust evidence of tissue rejuvenation [74], implicating young EVs as rejuvenating messengers of heart aging. HUCMSCs and their EVs are rich in youthful bioinformation and also play an important role in cardiac aging and related diseases.

Myocardial infarction and cardiac ischemia–reperfusion injury (IRI) models are widely used to validate the cardioprotective effects of HUCMSCs and their EVs in cardiac tissue repair and pathological post-injury remodeling. Generally speaking, the extraordinary abilities to inhibit cardiomyocyte apoptosis, regulate inflammatory responses, modulate oxidative stress, and enhance angiogenesis possessed by HUCMSCs and their EVs pave the way for their beneficial effects on repair and regeneration after myocardial infarction. HUCMSCs and their secretome have been shown to inhibit multiple forms of cardiomyocyte death in different contexts [75,76,77], and were validated to effectively alleviate lipid deposition and improve myocardial fibrosis and cardiomyocyte hypertrophy [78], accompanied with revascularization [79]. Equally, EVs released by HUCMSCs via paracrine mechanisms exert cardioprotective effects in a multifaceted manner. Given the limited regeneration capacity of differentiated cardiomyocytes with cycle arrest, current approaches concentrate on inducing the proliferation of existing cardiomyocytes through cell-cycle reentry [80]. The signaling cascade of circASXL1/CDK6/Rb1/cell-cycle reentry and circASXL1/Ncl/Ribo-bio/cytokinesis plays important roles in cardiac repair. Strikingly, HUCMSC-EVs were confirmed to coordinate the circASXL1-mediated signaling of cell-cycle reentry and Ribo-bio/cytokinesis to promote proliferation of cardiomyocytes and enhance cardiac repair [81]. In response to the pathological myocardial remodeling process, a study identified a novel mechanism whereby HUCMSC-EVs ameliorate myocardial hypertrophy through DJ-1, and this effect was particularly observed in EVs isolated from hypoxia-conditioned HUCMSCs [82]. In addition, miRNAs harbored in HUCMSC-EVs contribute to ameliorating inflammation, restoring myocardial fibrosis and promoting angiogenesis, including miR-223 [83], miR-136 [84], and miR-24 [85,86]. Amidst these therapeutic miRNAs, exosomal miR-136 derived from HUCMSCs demonstrated an anti-senescence effect in aged bone marrow MSCs and indirectly improved their capacity for myocardial repair by negatively regulating Apaf1 [84]. Likewise, HUCMSC-EVs also exert anti-senescence and cardio-protective effects by delivering circHIPK3, manifested by lower levels of p21, longer telomere length, and better cardiac function. At a mechanistic level, circHIPK3 serves as a scaffold facilitating the binding of p21 mRNA-binding protein HuR and the E3 ubiquitin ligase β-TrCP, leading to the degradation of HuR and reduction of p21 activity [87]. In another study, injection of HUCMSC-EVs encapsulated in hydrogel into the infarcted border zone of rat hearts improved myocardial function by reducing inflammation, fibrosis, and apoptosis, while promoting angiogenesis [88]. Interestingly, a recent study pointed out that MSCs isolated from different segments of the same umbilical cord had different regenerative properties, and maternal segment-derived HUCMSCs were a superior cell source for regenerative repair after myocardial infarction [89], implying the necessity to select the appropriate segment of umbilical cord as the source of EVs. In conclusion, HUCMSCs and their EVs present a unique value in improving myocardial hypertrophy, myocardial fibrosis, and other aging-related structural remodeling.

### 3.3. Effect of HUCMSC-EVs on Vascular Aging

Vascular aging is defined as structural and functional exacerbation with a high risk of spawning a range of ischemic or hemorrhagic disorders. Age-related vascular degradation increases vulnerability to atherosclerosis and ensuing cardio-cerebrovascular complications, which account for the majority of deaths worldwide [90]. However, effective interventions to counteract vascular aging remain to be discovered despite ongoing research efforts.

HUCMSCs and their EVs hold great promise as therapeutic agents for a series of vascular aging-induced disorders. HUCMSC-EVs have been demonstrated to prevent ischemic pathologies induced by age-related vascular dysfunction, and it was further found that miR-675 delivered by HUCMSC-EVs inhibited the TGF-β1/p21 signaling pathway to promote blood perfusion in ischemic hindlimbs [91]. Atherosclerosis represents a frequent complication of vascular aging, fueled by lipid-rich and inflammatory plaques accumulating medium-sized and large arteries [92]. The effect of stem cell-based therapy on atherosclerotic plaque formation and lesion progression has been explored and it is surprising that HUCMSCs treatment improved the early peripheral blood filling, reduced the serum lipid level, inhibited inflammatory responses, and elevated plaque stability by modulating the intestinal flora dysbiosis caused by the high-fat diet [93,94]. Based upon the existing literature, it is evident that HUCMSC-EVs exert a potent effect in enhancing angiogenesis and restoring pathological conditions of atherosclerosis. To be specific, the senescence of vascular endothelial cells (VECs) and vascular smooth muscle cells (VSMCs) tends to be the chief culprit of atherosclerosis [49]. Nanovesicles extracted from HUCMSCs were revealed to inhibit TNF-α-induced NF-κB activation to attenuate the endothelial inflammatory response and decrease the production of proinflammatory cytokines [95]. Considering loss of VSMCs in the medial layer of the thoracic aorta characterizing cardiovascular degradation during aging in mice [96], our previous study found a significant increase in the number of VSMCs and enhancement of angiogenesis in aged mice treated with HUCMSC-EVs [97]. Moreover, HUCMSC-EVs increased miR-100-5p in eosinophils, which acted on the FZD5/Wnt/β-catenin pathway to inhibit cell migration and inflammatory responses, thereby alleviating the progression of atherosclerosis [98]. Of note, atherosclerosis itself is rarely fatal, while it is thrombosis, superimposed on a ruptured atherosclerotic plaque, that results in life-threatening cardiovascular and cerebrovascular complications [92,99]. As stated before, the therapeutic capacity of HUCMSC-EVs for cardiac repair after myocardial infarction has been discussed. Similarly, HUCMSC-EVs are competent to enhance vascular endothelial remodeling and recover neurological function in rats suffering from ischemic stroke [100] and intracerebral hemorrhage [101]. HUCMSC-EVs-carried miR-24 could target AQP4 to activate the p38MAPK/ERK1/2/P13K/AKT pathway to decrease the infarct size, brain tissue apoptosis, and inflammation in rat models with cerebral ischemia reperfusion injury [102]. Altogether, the therapeutic efficacy of HUCMSC-EVs in vascular aging and related vascular diseases has been by far preliminarily confirmed. Further exploration on the role of HUCMSC-EVs in preventing the development of thrombosis-prone plaques and thus protecting the elderly population from fatal complications is of great interest.

### 3.4. Effect of HUCMSC-EVs on Kidney Aging

The kidney is a metabolically active organ that exhibits a gradual deterioration of structure and function during aging [103]. Kidney aging is characterized by the histological changes such as glomerular sclerosis, tubule atrophy, sparse capillaries, and interstitial fibrosis, as well as a progressive decrease in glomerular filtration rate and increase in the level of serum creatinine and blood urea nitrogen [104].

Small EVs derived from primary fibroblasts from young human donors effectively downregulate renal senescence markers in aged mice [105], implicating that young EVs play a rejuvenating role in aged kidneys. In line with this, intravenous injections of HUCMSC-EVs were found to reduce blood urea nitrogen and plasma creatinine levels in aged mice, and led to a marked decrease in the glomerular area, tubular atrophy, and interstitial fibrosis [97]. A recent study demonstrated that HUCMSC-EVs mitigated cellular senescence in the naturally aging kidneys. The researchers identified 24 proteins significantly differentially phosphorylated by the intervention of HUCMSC-EVs and found that phosphorylation of specific Lamin A/C sites was essential for anti-senescence [106]. Renal fibrosis is a canonical histological alteration of renal aging and an end result of pathological remodeling, caused by the trans-differentiation of renal tubular epithelial cells, fibroblasts, macrophages, etc., into myofibroblasts to produce an excessive extracellular matrix [107]. Notably, exosomal miR-186-5p and miR-335-5p from HUCMSCs were found to attenuate the inflammation and epithelial–mesenchymal transition (EMT) phenotype [108,109], providing a potential therapeutic strategy for renal fibrosis. Moreover, HUCMSCs have been reported to reduce the EMT of tubular epithelial cells in a diabetic nephropathy model by secreting EVs to inhibit the Hedgehog/SMO signaling pathway [110]. In addition, HUCMSC-EVs significantly reduced macrophage infiltration and downregulated the number of α-SMAs and F4/80 double-positive cells, suggesting that inhibition of the macrophage–myofibroblast transformation (MMT) process. In addition, aryl hydrocarbon receptor nuclear translocator-like (ARNTL, often referred to as Bmal1), is a central component of the circadian clock mechanism and was also proposed as a potential target of HUCMSC-EVs in hindering MMT and consequently preventing fibrosis [111]. In conclusion, HUCMSC-EVs show great therapeutic effect in renal age-related degeneration, especially renal fibrosis, and therefore are expected to evolve into promising anti-aging therapies for renal degenerative diseases.

Age-related renal degeneration augments the incidence and mortality of acute kidney injury (AKI) and chronic kidney disease (CKD) in the elderly [112,113]. A burgeoning body of research has suggested that HUCMSC-EVs played a crucial role in renal regeneration after injury caused by drugs and ischemia and abnormal structural alterations. For example, HUCMSC-EVs offer nephroprotective effects in drug-induced nephrotoxicity injury in both in vivo and in vitro models, which may be ascribed to ameliorating oxidative stress and inducing autophagy [114,115,116]. Furthermore, the expressions of senescence indicators such as LaminB1, p53, p21, p16, and senescence-associated β-galactosidase in tubular epithelial cells were significantly upregulated during renal IRI. Kidney subcapsular injection of HUCMSC-EVs improved renal function, reduced the level of senescent markers, and attenuated interstitial fibrosis in a mouse IRI model by regulating Bax/Bcl-2-dependent apoptosis and Ras-pERK-Ets1-p53 pathway-dependent cellular senescence [117]. Similarly, it has been highlighted that HUCMEC-EVs ameliorated ischemia-induced AKI and promoted tubular repair by targeting the cell-cycle arrest and apoptosis of tubular epithelial cells through the miR-125b-5p/p53 pathway [118], and by inhibiting endoplasmic reticulum stress via miR-148b-3p [119]. Intriguingly, HUCMSC-EVs exhibit preferential tropism to the ischemic kidney and prominently accumulate in proximal tubules by means of VLA-4 and LFA-1 on their surface [118]. Additionally, HUCMSC-EVs reversed renal injury induced by partial bladder outlet obstruction, and suppressed the Wnt/β-catenin signaling pathway [120], which interacts with the Notch, RAS, TGF-β/Smad, or Hedgehog/Gli signaling pathways to mediate age-related renal fibrosis [121].

In the context of CKD, the progressive decline of renal function precipitates an expedited aging trajectory, leading to premature aging as well as a disconnect between biological and chronological age [103]. CKD is therefore considered a disease associated with accelerated or premature aging. The available literature supports the fact that HUCMSC-EVs have showcased superior therapeutic effects in multiple preclinical models of CKD. In unilateral ureteral obstruction (UUO) mice, HUCMSC-EVs inhibited necroptosis and maintained mitochondrial functional homeostasis of injured renal tubular epithelial cells by delivering miR-874-3p to target RIPK1 and PGAM5, respectively [122], and were also reported to ameliorate renal fibrosis through CK1δ/β-TRCP-mediated YAP degradation and inhibition of the ROS-mediated p38MAPK/ERK signaling pathway [123,124]. In the disease model of diabetic nephropathy (DN), a leading cause of CKD [125], intravenous injection of HUCMSCs altered the expression of senescence-associated genes (p16, p53 and p21) and autophagy-associated genes (Beclin-1, p62, and LC3) by acting on the AMPK/mTOR pathway through a paracrine action mode, protecting the kidney from diabetic damage [126]. Research has shown that HUCMSC-EVs decreased the production of pro-inflammatory cytokines and pro-fibrotic factors in high glucose-injured renal tubular epithelial cells and glomerular endothelial cells [127]. Similarly, HUCMSCs are recognized as a novel antifibrotic modality for the treatment of DN due to the inhibition of renal fibrotic alterations by targeting the Hedgehog/SMO signaling pathway through the release of EVs [110]. In addition, peritoneal dialysis is one of the alternative treatments for CKD patients, and peritoneal fibrosis due to EMT induced by chronic stimulation of dialysate is one of the major causes of ultrafiltration failure. Yang and colleagues have demonstrated that HUCMSC-EVs could alleviate the EMT of peritoneal mesothelial cells by transferring lincRNAs (CDHR, GAS5) [128,129]. Overall, HUCMSC-EVs exhibit a prominent effect in restoring the functional decline and structural destruction of aging kidneys, and are expected to improve the clinical outcomes of AKI and CKD.

### 3.5. Effect of HUCMSC-EVs on Muscle Aging

Age-related sarcopenia is a progressive and generalized loss of muscle mass, strength, and function, and gives rise to a wide assortment of adverse outcomes including falls, frailty, and mortality [130,131]. Currently, there is no efficient treatment to prevent and reverse age-related fragility, and the search for therapies to ameliorate sarcopenia is of primary concern in the field of muscle aging.

Circulating EVs from young mice are determined to rejuvenate aged cell bioenergetics and promote skeletal muscle regeneration [26]. HUCMSCs have been shown to recover impaired muscle vitality and function during aging due to their capacity to activate resident muscle satellite cells, enhance autophagy, combat cellular senescence, inhibit apoptosis, alleviate chronic inflammation, improve mitochondrial function, and upregulate myogenic factors expression levels [132,133,134]. With similar functions, HUCMSC-EVs are postulated to serve as a novel therapeutic tool for muscle regeneration. The potential of HUCMSC-EVs for the treatment of muscle atrophy induced by diabetes, obesity, and nerve injury has been well evaluated [135,136], which may be related to their autophagy regulatory properties by targeting Fbxo32 and Trim63 [136]. The study performed by Ma et al. demonstrated the remarkable capacity of HUCMSC-EVs to mitigate muscle atrophy in age-related and dexamethasone-induced sarcopenia mice models via the miR-132-3p/FoxO3 axis, providing valuable insight into the utilization of HUCMSC-EVs for the treatment of sarcopenia [137]. Consistent with the above, HUCMSC-EVs released circHIPK3 to prevent pyroptosis by targeting the miR-421/FOXO3a axis under the circumstance in skeletal muscle ischemic injury [138]. These findings accentuate a promising strategy for aging-induced muscle diseases and consolidate the evidence for the clinical transformation potential of HUCMSC-EVs for sarcopenia. Given the contributions of muscle satellite cells made to recovering muscle function after injury, further investigation is entailed to decipher whether HUCMSC-EVs can counteract senescence-related phenotypes of muscle satellite cells and restore their number and function in aged muscle.

### 3.6. Effect of HUCMSC-EVs on Skeletal Aging

Bone aging, characterized by decreased bone density, weakened internal architecture, and increased vulnerability to fracture [139], is essentially an imbalance between bone formation and bone resorption and constitutes a major public health concern. In the prior research, we have observed an increase in trabecular mass and trabecular area of the distal femur, along with a decrease in osteoclasts and an increase in osteogenesis, in HUCMSC-EV-treated 16-month-old mice [97]. Another work revealed that HUCMSC-EVs could effectively promote proliferation, migration, and osteogenic differentiation of a mouse cranial preosteoblast cell line [140]. It should be noted that the senescence of bone marrow MSCs is one of the key factors of bone aging. Senescent bone marrow MSCs show a stronger tendency towards adipogenic differentiation, leading to decreased bone formation accompanied by accumulation of adipose tissue in bone marrow [141,142,143]. Intriguingly, HUCMSC-EVs rejuvenate senescent adult bone marrow MSCs, increasing telomere length and elevating their self-renewal capacity [97]. On this basis, HUCMSC-EVs have a significant impact on the direction of osteogenic/adipogenic differentiation of bone marrow MSCs. Evidence suggests that CLEC11A (c-type lectin structural domain family 11 member A), with high therapeutic potential in treating a variety of bone disorders [144], is highly enriched in HUCMSC-EVs, and thus enhances the shift from adipogenic to osteogenic differentiation of bone marrow MSCs [145]. It is worth noting that the bone regenerative effect of HUCMSC-EVs may not be entirely attributed to the influence on the differentiation of bone marrow MSCs, as HUCMSC-EVs have been found to contribute to osteogenesis and accelerate fracture healing by promoting endothelial cell viability and angiogenesis [146,147]. Paradoxically, the former research result demonstrated that HUCMSC-EVs did not affect the osteogenic differentiation of bone marrow MSCs [146].

Osteoporosis is a progressive disorder of diminished bone mass and microarchitectural deterioration, and poses a high risk for fragility fractures with aging [148]. Age-related osteoporosis is evident by the decreased average bone wall thickness and number of cells in the bone marrow and the increased adipose tissue [49]. The incidence of osteoporosis and osteoporotic fractures increases with age and is particularly pronounced in the postmenopausal female population. Therefore, the main model used in mice to study osteoporosis is the induction of postmenopausal osteoporosis through ovariectomy in female mice [149]. HUCMSCs are expected to alleviate osteoporosis when transplanted in vivo. These effects include improving bone structure, increasing bone density and the number and circumference of trabeculae, and enhancing osteogenic differentiation of bone marrow MSCs, achieved by activating the WNT/β-catenin pathway and inhibiting inflammation [150,151,152]. The capacity of secretome from HUCMSCs to restore stem cell potential and delay local bone loss in age-related osteoporosis has been reported [153]. As an important part of the secretome, HUCMSC-EVs also have therapeutic value in improving the phenotypes of osteoporosis models. As previously described, HUCMSC-EVs ameliorate bone loss and enter bone marrow cells in aged mice while enhancing osteogenesis and attenuating adipogenesis in adult bone marrow MSCs in vitro [97]. Deluca et al. demonstrated that a single, local application of HUCMSC-EVs in combination with a low-dose recombinant human rhBMP-2 played a synergistic role in the regeneration of osteoporotic bone defects [154]. Moreover, hypoxic preconditioning activates HIF-1α in HUCMSC to mediate enhanced production of exosomal miR-126 for bone fracture healing [155]. Ge et al. analyzed the effect of osteogenic induction on the functions of HUCMSC-EVs. Specifically, exosomal miRNAs from osteogenesis-induced HUCMSCs are involved in bone differentiation and formation; the expression of hsa-mir-2110 and hsa-mir-328-3p gradually increased with the prolonged osteogenic differentiation and regulated target genes associated with bone differentiation, indicating the importance of osteogenic regulatory miRNAs in HUCMSC-EVs [156]. In conclusion, the beneficial effects of HUCMSC-EVs on osteoporosis and bone fracture repair may be mainly attributed to their anti-inflammatory activity and contribution to osteogenic differentiation of bone marrow MSCs. However, how HUCMSC-EVs act on osteoblasts and osteoclasts to alter their viability remains unclear, and the key molecules that determine the differentiation of bone marrow MSCs affected by HUCMSC-EVs need to be further studied.

Osteoarthritis (OA) is a prevalent degenerative joint disease primarily affecting hyaline cartilage with a characteristic feature of progressive cartilage degradation and synovial membrane inflammation [157]. OA accounts for a major cause of adult disability and there is no effective treatment to halt its progression [158]. EVs from young human plasma were detected to effectively stimulate chondrocyte proliferation and migration, and more importantly, the benefits can be maintained in the inflammatory microenvironment [19]. By the same token, intra-articular or systemic injection of HUCMSCs and their EVs is an appealing strategy in regenerative medicine due to their immunomodulatory properties that combat excessive inflammation in OA. The safety and efficacy of HUCMSCs therapy for OA have been demonstrated in clinical and preclinical studies [159,160,161,162,163]. Further, a clinical study of 30 patients with knee osteoarthritis showed that intra-articular injection of HUCMSC secretome demonstrated superior clinical improvement, biomarker changes, with no adverse effects over a 5-week interval [164]. EVs derived from HUCMSCs have exhibited their remarkable potential as a therapeutic agent to rewire the cartilage microenvironment in preclinical OA animal models established by surgery and intra-articular injection of different toxins (iodoacetic acid, IL-6, etc.). Injection of HUCMSC-EVs in the articular lumen could promote the polarization of M2 macrophage to inhibit inflammation levels in OA chondrocytes, thereby improving the progression of joint lesions in OA models [165]. Yang et al. found that the levels of inflammatory cytokines in the synovial fluid of a HUCMSC-EV treatment group were lower than those of the control group. The expression of MMP-13 and ADAMTS-5 was downregulated while collagen II was upregulated in chondrocytes [166]. Concomitantly, miR-1208 and miR-223, enriched in HUCMSC-EVs, are suggested to act on downstream inflammatory factor (e.g., NLRP3) gene expression to exert anti-inflammation and chondroprotective effects [167,168]. Similarly, apoptotic vesicles released by transplanted HUCMSCs also triggered M2 polarization in macrophages and facilitated the chondrogenic differentiation of bone marrow MSCs, mediated by miR-100-5p/MAPK/ERK and let-7i-5p/eEF2K/p38 MAPK, respectively [169]. Collectively, EVs, as secretory components of HUCMSCs, hold anti-inflammatory and chondroprotective promise for OA with safety guaranteed. However, it should be noted that etiological factors of OA are joint specific [170], but the majority of the current findings are in knee OA models. The therapeutic efficacy and promise of HUCMSC-EVs in other joints and their mechanisms need to be further studied in the future.

### 3.7. Effect of HUCMSC-EVs on Endocrine Pancreatic Aging

The endocrine part of the pancreas secretes a variety of hormones to maintain the homeostasis of the blood glucose balance. β cells make up 60–80% of the islet cell population and the age-related change in islet cells accounts for the strong association of type 2 diabetes (T2D) incidence with age [49]. Strikingly, in a diabetic rat model, HUCMSCs not only improve blood glucose, but also protect the vascular endothelium from diabetic damage, in which MAPK/ERK signaling mediated its molecular mechanism of paracrine action, thus providing a novel insight into the clinical treatment of diabetes and its vascular complications [171]. HUCMSC-EVs hold promise as a cell-free therapy for the treatment of T2D. The work performed by Sun et al. has confirmed that HUCMSC-EVs could not only inhibit streptozotocin-induced apoptosis of β cells and restore insulin secretion function, but also promote the membrane translocation of glucose transporter 4 in muscle and increase glycogen storage in liver to partially reverse insulin resistance [172]. Xia et al. observed that the therapeutic effects of HUCMSC-EVs in T2D mice manifested through the reduction of random blood glucose levels, enhancement of glucose and insulin tolerance, and increased insulin secretion, which were achieved by the exosomal delivery of active proteins abundant in the AKT/ERK pathways to inhibit NRF2-mediated ferroptosis to augment β cell mass [173]. Devastating macrovascular complications (cardiovascular disease) and microvascular complications (such as diabetic kidney disease, diabetic retinopathy, and neuropathy) spawn increased mortality, blindness, kidney failure, and an overall decreased quality of life in individuals [174]. Table 1 summarizes the recent research findings about the therapeutic effects of HUCMSC-EVs on various complications of diabetes mellitus (diabetic nephropathy has already been mentioned in the previous section). Overall, HUCMSC-EVs showcased a stark therapeutic potential of diabetic complications due to their capability of angiogenesis, anti-inflammation, anti-apoptosis, autophagy modulation, and interplay with immune cells properties.

### 3.8. Effect of HUCMSC-EVs on Aging of Other Organs

HUCMSC-EVs also play a key role in delaying aging and repairing damage in the liver, skin, ovary, and testis. The relevant literature has been summarized in Table 2.

## 4. Clinical Studies of HUCMSC-EVs for Human Age-Related Diseases

As discussed above, the therapeutic efficacy of HUCMSC-EVs in preclinical models has been extensively validated, and it is also worth noting that clinical studies related to HUCMSC-EVs are also in full swing. According to the clinical trials registered in ClinicalTrials.gov (accessed on 24 November 2024), it is evident that HUCMSC-EVs have garnered significant attention for their potential in addressing a wide range of pathologies that often lack adequate treatment options, including but not limited to COVID-19 pneumonia (NCT05787288), androgenic alopecia (NCT06697080), neonatal diseases (NCT06279741), decompensated liver cirrhosis (NCT05871463) and amyotrophic lateral sclerosis (NCT06598202).

Our prime focus is on the clinical application of HUCMSC-EVs for the treatment of age-related diseases. In a single-center, randomized, placebo-controlled clinical study, 20 patients with stage III and IV CKD who received HUCMSC-EVs treatment via the intra-arterial route exhibited significant improvements in renal function indicators. Compared with the placebo group, participants had significantly higher plasma levels of TGF-β1 and IL-10, as well as significantly lower plasma levels of TNF-α [216]. However, the age range of enrolled patients was 26 to 44 years, and elderly patients were not included in this trial. An ongoing clinical trial led by Pinlei Lv (NCT06495437) aims to evaluate the initial safety of HUCMSC-EV infusion in patients with age-related impaired glucose tolerance. In this single arm study, eligible subjects are scheduled to receive 100 mL HUCMSC-EV formulation intravenously over a period of 1–1.5 h. Further observation of the intervention’s efficacy and safety will continue for an additional 3 months following the final follow-up visit. A multi-center, open, single-arm, basket-design clinical trial led by Hao et al. (NCT06607900) is intended to evaluate the safety and preliminary efficacy of HUCMSC-EVs nasal drops in multiple neurodegenerative diseases, including AD, PD, and frontotemporal dementia. Additionally, in a clinical experimental study targeting OA (NCT06688318), researchers aim to explore the effectiveness of intra-articular injections of conditioned media from HUCMSCs to knee joint OA patients with age over 45 years, followed by assessments using functional scores and MRI with T2 mapping sequence.

Thus, clinical research involving HUCMSC-EVs remains in a relatively nascent stage. Ongoing pilot studies assessing the safety and efficacy of HUCMSC-EV-based therapies have provided preliminary insights into their application in human subjects; however, these studies also underscore the challenges that must be tackled to ensure the successful clinical translation. Notable knowledge gaps persist in optimizing isolation and purification protocols, achieving standardized large-scale production, exploring administration approaches, and understanding the biological distribution and clearance of HUCMSC-EVs [20,217]. In addition, establishing placebo controls will be essential for future studies, as this would provide more solid evidence to substantiate the efficacy of HUCMSC-EVs.

## 5. Engineered HUCMSC-EVs for Anti-Aging Therapies

Despite the crucial role of natural HUCMSC-EVs in slowing down age-related changes in the organism, challenges including low yield, insufficient targeting property, and inadequate therapeutic efficiency still hinder the clinical application of HUCMSC-EVs to treat age-related diseases. As a consequence, researchers are actively engaged in surmounting hurdles through engineering approaches for optimized therapeutic strategies.

### 5.1. Refinement of Culture Conditions for Increased Production of HUCMSC-EVs

Producing a sufficient amount of EVs for therapy and therapeutic applications remains challenging. Conventional cellular culture and secretion is unable to generate the quantities that meet clinical dosage needs. Considerable effort is being expended to improve the yield of HUCMSC-EVs. Here, we discuss the strategies to improve EV yield and alter EV cargo compositions by adjusting the culture conditions of HUCMSCs culture.

Light stimulation is acknowledged as a simple, straightforward, low-cost, non-invasive, and generic method for promoting a variety of cell types [218]. Ruan et al. achieved more than a 13-fold enhancement in the dendritic cell-derived EV production rate via light promotion under the optimal intensities and exposure times [218]. The optimized light parameters (wavelength, intensity, area, time, etc.) will facilitate the growth of MSCs and the release of their EVs. A study by Zhang et al. demonstrated that light induction effectively boosted the proliferation and alleviated the cellular senescence of adipose-derived MSCs, which exhibited a fivefold increase in EV secretion after only 30 min of light exposure [219]. However, at present, there are no reports showing that light treatment can improve the yield of HUCMSC-EVs, and the optimal parameters for HUCMSC culture have not been explored yet.

Extracellular matrix (ECM) is a complex network of proteins and carbohydrates surrounding cells, which provides physical cues that influence cellular behaviors including adhesion, migration, and differentiation [220]. Consistent with this, HUCMSCs tend to differentiate directly towards cardiomyocytes on the suitable matrix stiffness (13–16 kPa) [221]. Particularly, the mechanical properties of ECM have a profound impact on EV biology. For instance, a rigid ECM can promote EV secretion from tumor cells [222,223]. Further, bone marrow MSCs secrete 10 times more EVs on a soft matrix than on a rigid one [224]. In view of this, Liu et al. recently demonstrated that ECM stiffness had a notable effect on the components of HUCMSC-EVs, which in turn affected the sorting of lipids and proteins into EVs. Multi-omics analysis indicated that ECM stiffness could alter protein fractions in EVs by affecting the protein membrane localization, leading to higher effectiveness in targeting macrophages [225]. Hitherto, there is no robust evidence supporting the effect of mechanical properties of ECM on EV biology derived from HUCMSCs, but relevant studies are of prime interest.

Three-dimension (3D) aggregation, as a preconditioning strategy, is expected to promote MSC-EV production with a beneficial cargo profile compared to 2D culture [226]. In line with this, cultivation of HUCMSCs in scalable microcarrier-based 3D cultures combined with conventional ultracentrifugation yields 20-fold more EVs than 2D cultures. Furthermore, tangential flow filtration in combination with 3D HUCMSC cultures further increased the yield of EVs sevenfold more than only 3D cultures [227]. Using hollow-fiber bioreactors to achieve a 3D culture of HUCMSCs, studies performed by Yan et al., Sun et al. and Cao et al. achieved 7.5-fold, 8.2-fold, and 19.4-fold increases in EV production, respectively, and demonstrated that 3D-cultured HUCMSC-derived EVs had more potent therapeutic potential for cartilaginous, cardiac, and renal injuries [228,229,230]. Moreover, HUCMSCs were 3D cultured using an ultra-low attachment surface method, a total EV yield of which was also upregulated (5.6-fold). EVs derived from 3D-cultured HUCMSCs significantly rescued the cell viability of cyclophosphamide-treated ovarian granulosa cells [231]. Additionally, in order to exclude the side effects of serum-derived impurities, Kim et al. introduced 3D culture and cell-priming approaches for HUCMSCs in serum-free chemically defined media. Under this condition, the secretion of HUCMSC-EVs was significantly enhanced about 6.7 times by aggregates (spheroids) with a 3D culture than cells with a 2D culture. Further modulation with cell priming with the combination of TNF-α and IFN-γ facilitated the production of HUCMSC-EVs about 1.4 times more than cells without priming (9.4 times more than cells with 2D culture without priming [232]. These findings support the idea that the 3D culture of HUCMSCs may contribute to the mass production of their EVs, thus, further expanding their value in regenerative medicine.

### 5.2. Surface Modification for Enhanced Targeting Property of HUCMSC-EVs

Surface modification contributes to precisely targeting HUCMSC-EVs to specific senescent cell types or tissues, which is critical for targeted anti-aging therapeutic interventions. Lentiviral transfection and chemical coupling modification were used to conjugate targeting ligands or specific molecules on the surface of HUCMSC-EVs [233]. The achievement of selectively transported goods to intended sites is particularly beneficial to minimizing off-target effects, offering customized treatments with higher efficacy and safety.

Genetic engineering involves genetically modifying HUCMSCs to overexpress therapeutic biomolecules. Our recent study applied the principle of CAR-T targeted antitumor therapy to engineering HUCMSC-EVs as more targeted anti-aging biotherapeutics. Specifically, we constructed CD38 antigen receptor membrane-modified HUCMSC-EVs by transfecting MSCs with a lentivirus loaded with a CD38 antigen receptor−CD8 transmembrane fragment fusion plasmid to target and rejuvenate senescent type 2 alveolar epithelial cells, which express a high level of CD38 (a marker of senescent cells) and play a pivotal key role in age-associated pulmonary fibrosis [234]. Similarly, Liu et al. genetically engineered a collagen II-targeting peptide onto the surface of HUCMSC-EVs, achieving a more targeted and efficient delivery of miR-223 with chondroprotective effects [168]. In the study performed by Ji et al., a higher renal-targeting antifibrotic therapeutic effect was achieved by utilizing superparamagnetic iron oxide nanoparticles (SPION) modified HUCMSC-EVs with a high expression of carboxyl terminus of Hsc70-interacting protein via lentiviral transduction [235]. HSTP1, an identified targeting peptide to activated hepatic stellate cells, was fused with exosomal-enriched membrane protein (Lamp2b) and was displayed on the surface of HUCMSC-EVs through genetic engineering technology, leading to an enhanced therapeutic effect on liver fibrosis [236]. Notably, recruiting molecules (Tetraspanins, Lamp2b, vesicular stomatitis virus G, etc.) with high EV-sorting ability should be taken into consideration when constructing the vector [237,238]. In practical terms, researchers can genetically modify HUCMSCs to overexpress the target molecules fused with a recruiting molecule, facilitating their incorporation into EVs [239]. Targeting-mediated molecules with different molecular weights may need to be fused with different recruiting molecules, which needs to be analyzed on a case-by-case basis.

Meanwhile, chemical modification ensures that the biological activity and stability of EVs are not compromised while achieving the desired engineered characteristics [233]. Cao et al. engineered HUCMSC-EVs with the designed chondrocyte-targeting polymer (chondrocyte affinity peptide) on the membrane, and the enrichment of engineered HUCMSC-EVs on an articular cartilage surface was more than three times that of natural HUCMSC-EVs [240]. In addition, Zhang et al. designed biomimetic nanovesicles from HUCMSCs surface engineered with cardiac-targeting peptide. With the help of the myocardial targeting effect of homing peptides, nanovesicles can be enriched in the lesion site to exert a more efficient cardioprotective function [241]. According to Xia et al., Michael addition reaction-mediated chemical coupling was used to modify the surface of the EV membrane with a β-cell-targeting aptamer and polyethylene glycol, which improved the pancreatic islet β-cell targeting of HUCMSC-EVs and protected pancreatic islets with greater precision and efficiency [173].

Of note, surface modification can improve the targeting property and bioavailability of HUCMSC-EVs. However, surface modification requires precise chemical or biological reaction conditions, otherwise it may alter the HUCMSC-EVs’ natural properties and trigger an immune response or other adverse reactions.

### 5.3. Cargo Loading for Enhanced Anti-Aging Effect of HUCMSC-EVs

EVs are opening new frontiers for modern drug delivery. Cargo loading involves incorporating therapeutic molecules or drugs into EVs through multiple methods to achieve the purpose of drug delivery [233].

For example, EVs purified from HUCMSCs transfected with miR-211-5p inhibitor plasmid exhibited significantly greater efficiency than control EVs in AD treatment by increasing NEP expression [62]. Researchers encapsulated docetaxel into EVs derived from HUCMSCs transfected by miR-125a by optimized mild sonication-incubation technique, demonstrating prominent anti-metastatic behavior [242]. Another study obtained engineered HUCMSC-EVs by transfecting parental cells with miR-126-3p lentivirus vector to achieve a superior pro-angiogenesis and anti-apoptosis effect in a preclinical rat model of premature ovarian failure [197].

Actually, we found that the studies concerning cargo loading of HUCMSC-EVs mainly focused on miRNAs, and most of them were achieved via genetic engineering. Of note, some large molecule drugs can also be loaded into EVs from other cell types to improve their stability and bioavailability [243], but more evidence remains to be provided to confirm whether it is generic for HUCMSC-EVs. The feasibility of methods including electroporation, extrusion, sonication, and incubation for cargo loading into HUCMSC-EVs are also worth further exploring. Undeniably, the precise control of the type and dose of therapeutic substances encapsulated in EVs will provide insight into a more efficient therapeutic modality.

## 6. Conclusions and Future Perspectives

Analogous to circulating EVs in the young blood, HUCMSC-EVs are emerging as promising agents in the advancement of anti-aging therapeutics. Characterized by superior accessibility and safety, HUCMSC-EVs have a marked capacity to alleviate inflammation, inhibit apoptosis, modulate autophagy, regulate mitochondrial function, and promote regeneration, thus being regarded as a multi-target therapeutic tool for organismal aging and age-related diseases. Predicated on this, a growing number of studies delved into developing novel strategies to HUCMSC-EV engineering by increasing the yield, enhancing senescent cell-targeting properties, and further amplifying their anti-aging effect.

However, the molecular mechanisms underlying the anti-aging effects of HUCMSC-EVs are largely unknown. It is urgent to uncover how HUCMSC-EVs rewire established pro-aging signatures to activate pro-youth gene sets by conducting comprehensive and systematic nucleic acid and proteomic sequencing of the cargos harbored in different HUCMSC-EVs subsets. It should be emphasized that the aging animal models currently used to detect the anti-aging effect of HUCMSC-EVs are mostly induced by drugs, while the studies using natural aging models are still in a small minority, but the molecular mechanisms involved in each may be different. More importantly, leveraging computational tools and artificial intelligence is advocated to harness the full potential of single-cell data, which reveals the susceptibility of various cell populations in the organs to the impairment of aging and deciphers the molecular changes in cellular senescence. These results help us identify the key cells in different tissues that mediate the pathologies of age-related disorders, design engineered HUCMSC-EVs that act more efficiently on these cells, and then devise precise and effective anti-aging interventions. Although promising, there is no record of engineered HUCMSC-EVs being used in clinical trials. The heterogeneity of HUCMSCs and their EVs must be taken into account in order to accelerate the clinical translation of HUCMSC-EVs as a therapeutic agent for age-related disorders. For instance, segmental localization of the entire umbilical cord when isolating HUCMSCs, selection of parental HUCMSCs with appropriate viability, passage and culture conditions when collecting supernatant, and HUCMSC-EVs’ isolation and purification protocols must be standardized and uniformed. Furthermore, the clinical application of HUCMSC-EVs still needs to address the issue of yield and quality management [244,245]. In future research endeavors, it is necessary to further explore the normalized separation and purification of HUCMSC-EVs, while also assessing the potential side effects in the therapeutic context of age-related diseases, including but not limited to, thrombosis [246].

Overall, HUCMSC-EVs have exhibited a broad spectrum of efficacy in addressing age-related disorders in multiple organs through diverse mechanisms. We are convinced that both HUCMSC-EVs and their cargos are promising therapeutic tools for aging and age-related diseases, thanks to their ability to modulate multiple characteristics linked with the aging process. There are ongoing efforts to engineer HUCMSC-EV as a more efficient anti-aging therapy, and thus unlock the full potential of these nanostructures.

## Figures and Tables

**Table 1:** Therapeutic efficacy of HUCMSC-EVs in diabetic complications.

Diabetic Complications	Models	Effects	Pathway Involved	Characteristics	Reference
Diabetic dermopathy	db/db and db/m mice with cutaneous wound	UCMSC-ApoVs improved skin defect healing, inhibited macrophage pyroptosis in vivo as well as in vitro, reduced oxidative stress levels	Not mentioned	Anti-oxidative stress Anti-pyroptosis	[175]
	STZ-induced SD rats with a full-thickness cutaneous wound	HUCMSC-EVs accelerated wound closure rate, increased expression of CD31 and Ki67, enhanced regeneration of granulation tissue, and upregulated expression of VEGF and TGF-β1	Increased VEGF and TGF-β1	Angiogenesis	[176]
	STZ-induced SD rats with a full thickness skin wound	Treatment of HUCMSC-EVs enhanced migration and angiogenesis of HUVECs in vitro and demonstrated 93.3% wound closure ability, accompanied by a high degree of re-epithelialization	Not mentioned		[177]
	STZ-induced SD rats two full thickness skin wounds	HUCMSC-EVs played a positive role in wound closure rate, epidermal healing, and collagen deposit in vivo, and promoted proliferation and migration of fibroblasts in vitro	Not mentioned		[178]
	Diabetic C56BL/6N mice with a sterile 8 mm biopsy punch incision	HUCMSC-EVs increased the phosphorylation of STAT6 in most neutrophils and oriented neutrophils towards an anti-inflammatory N2 phenotype, which boosted the release of proangiogenic factors, particularly BV8, and induced angiogenesis	Activation of Jak2/STAT6 signaling pathway	Polarization of neutrophils Angiogenesis	[179]
	STZ-induced DM rats with a full-thickness excisional wound on the dorsal skin	HUCMSC-EVs promoted the proliferation of HUVECs and NIH-3T3 in vitro, induced M2 macrophage polarization, and reduced wound area and inflammatory infiltration and increased collagen fibers in vivo	Not mentioned	Anti-inflammation Angiogenesis Collagen Synthesis and deposition	[180]
	STZ-induced DM rats with full-thickness skin defect	HUCMSC-EVs integrated into the gallium/chitosan/silk solution facilitated angiogenesis, suppressed bacterial growth and inflammation, as well as promoted collagen deposition and re-epithelialization of wounds	Not mentioned	Angiogenesis Anti-infection Anti-inflammation	[181]
	Patients with diabetes, non-diabetic ulcers, and diabetic foot ulcers	HUCMSC-EVs transferred functional mitochondria to neutrophils in wound tissue, triggered mitochondrial fusion, and restored mitochondrial function, thereby reducing NET formation	Transfer of functional mitochondria	Mitochondrial homeostasis Inhibition of NET formation	[182]
Diabetic cardiomyopathy	High-fat, high-sugar diet mixed with STZ-induced DM rat	HUCMSC-EVs restored cardiac functions and reversed ventricular remodeling	Activation of AMPK-ULK1 signaling pathway	Autophagy	[183]
Diabetic retinopathy	STZ-induced C57BL/6J mice	HUCMSC-EVs shuffled miR-17-3p to reduce the blood glucose and HbAlc, increase the weight, Hb content, and GS level, and ameliorate the inflammatory reaction and oxidative injury of DR mice	miR-17-3p/STAT1	Anti-inflammation Anti-oxidative stress	[184]
	STZ-induced DM rat	HUCMSC-EVs alleviated retinal structure disruption and reduced the apoptosis of RGCs	phosphorylation of p38MAPK	Anti-apoptosis	[185]
	STZ-induced DM rat	HUCMSC-EVs prevented early retinal vascular damage and thickening of the retina and alleviated retinal structure disruption	Not mentioned		[186]
	STZ-induced DM rat HG-treated HRECs	HUCMSC-EVs decreased the inflammation reaction by transferring miR-30c-5p in DM rats and HG-treated HRECs	miR-30c-5p PKD/NF-κB	Anti-inflammation	[187]
	STZ-induced DM rat HG-treated hRMECs	HUCMSC-EVs reduced the level of vascular leakage in the retinas of rats but also decreased the retinal thickness as well as the associated inflammation in vivo and repressed high glucose-induced cell inflammation and apoptosis	MiR-18b/ MAP3K1/NF-κB axis	Anti-apoptotic Anti-inflammatory	[188]

Here, we present a comprehensive overview of the rejuvenating characteristics and therapeutic efficacy of HUCMSC-EVs in diabetic complications including diabetic wound healing, diabetic cardiomyopathy, and diabetic retinopathy. DM: diabetes mellitus; ApoVs: apoptotic vesicles; STZ: streptozotocin; VEGF: vascular endothelial growth factor; TGF: transforming growth factor; NET: neutrophil extracellular trap; HbAlc: glycosylated hemoglobin; Hb: hemoglobin; GS: glutamine synthetase; DR: diabetic retinopathy; RGCs: retinal ganglion cells; p38MAPK: p38 mitogen-activated protein kinase; HG: high glucose; HRECs: human retinal endothelial cells.

**Table 2:** Therapeutic effects of HUCMSC-EVs in age-related pathological conditions of multiple organs.

Organ	Model	Reference
Liver	Natural aging livers	[189]
	Hepatic ischemia reperfusion injury	[190,191,192,193,194,195]
Reproductive organs	POF	[196,197,198,199,200,201,202,203,204,205]
	Natural aging testis	[206]
Lung	COPD	[207]
	Papain-induced emphysema	[208]
	IPF	[209]
	Chronic asthma	[210]
Skin	Photoaging	[211,212,213,214]
	Skin aging and rejuvenation	[215]

Concise outline of the therapeutic effects of HUCMSC-EVs in age-related pathological conditions in the liver, ovary, testis, lungs, and skin. POF: premature ovarian failure; COPD: chronic obstructive pulmonary disease; IPF: idiopathic pulmonary fibrosis.
